# Microbiological and chemical characterization of coastal hospital wastewater in Oran, Algeria: Implications for public and livestock health under a One Health framework

**DOI:** 10.14202/vetworld.2025.1819-1830

**Published:** 2025-07-08

**Authors:** Sabrina Hannachi, Djillali Bouras, Roger Flower

**Affiliations:** 1 Department of Biology, Faculty of Natural and Life Sciences, University Oran 1 Ahmed Ben Bella, Oran, Algeria; 2 Department of Geography, University College London, United Kingdom

**Keywords:** Algeria, antibiotic resistance, *Escherichia coli*, hospital wastewater, one health, trace metals

## Abstract

**Background and Aim:**

Hospital effluents are a major source of environmental contaminants, harboring pathogenic bacteria, toxic trace metals, and high organic loads. This study aimed to evaluate the bacteriological and physicochemical profiles of wastewater discharged from three coastal hospitals in Oran, Algeria, and to assess the associated public and livestock health risks under the One Health approach.

**Materials and Methods:**

A cross-sectional study was conducted from January 2023 to February 2024, involving monthly sampling at three hospitals and one drainage collector. Twenty-six composite samples were collected at three peak daily intervals. Bacteriological analysis targeted *Escherichia coli, Staphylococcus aureus*, and *Salmonella* spp. using selective media, membrane filtration, and biochemical confirmation. Physicochemical parameters, including biochemical oxygen demand (BOD_5_), chemical oxygen demand (COD), dissolved oxygen (DO), pH, and conductivity, were analyzed using standard American Public Health Association methods. Trace metals (lead [Pb], cadmium, nickel, copper [Cu], zinc) were quantified through atomic absorption spectrometry.

**Results:**

All effluents contained pathogenic bacteria, with peak concentrations of *E. coli* (up to 34.5 × 10^6^ colony-forming units [CFU]/100 mL), *S. aureus* (up to 4.5 × 10^6^ CFU/100 mL), and persistent *Salmonella* spp. detected primarily in warmer seasons. All *S. aureus* and *Salmonella* isolates exhibited resistance to multiple antibiotics. Physicochemical assessment revealed elevated BOD_5 (_190 mg/L–398 mg/L), COD (200 mg/L–590 mg/L), and COD/BOD_5_ ratios <2.5, indicating high organic pollution with partial biodegradability. Trace metal concentrations, particularly Pb and Cu, exceeded the World Health Organization discharge guidelines in all samples. Contaminant levels were highest in summer, correlating with increased hospital activity and temperature.

**Conclusion:**

Untreated hospital wastewater in Oran poses a serious threat to public and environmental health. The presence of multidrug-resistant bacteria and toxic metals highlights the urgent need for dedicated hospital wastewater treatment infrastructure. Grazing livestock and marine ecosystems exposed to these effluents are at risk of bioaccumulation and infection. Regulatory enforcement, routine monitoring, and the implementation of sustainable green hospital plans are essential to safeguard health under the One Health paradigm.

## INTRODUCTION

Hospital effluent differs significantly from munici-pal wastewater due to its elevated concentrations of toxic substances, pathogenic microorganisms, and hazardous chemicals, all of which pose serious risks to both human and environmental health. Healthcare institutions consume large quantities of water during their operations, generating hazardous and medical waste that necessitates specialized treatment and disposal protocols. When improperly managed, such discharges contaminate both surface and groundwater resources [1, 2]. The World Health Organization (WHO) estimates that hospital operations require approxi- mately 50 L of water/patient/day, with surgical proce-dures consuming up to 100 L/intervention [3, 4]. In the case of patients with severe acute respiratory syndrome or viral hemorrhagic fevers, water require-ments can range between 100 L and 400 L/patient daily [4]. Consequently, hospital activities produce substan-tial volumes of wastewater [5, 6].

Several factors influence hospital wastewater volume, including bed capacity, facility size and type, available infrastructure, the scope of services offered (e.g., laundry, kitchen, sterilization), and internal wastewater management systems [7, 8]. As noted by Majumder *et al*. [2], daily per capita hospital wastewater production varies from 200 L to 400 L in developing countries to 400 L–1200 L in developed nations. The continuous release of untreated hospital effluents poses a global risk to aquatic ecosystems. Such wastewater typically contains pharmaceutical residues, pathogenic organisms, chemical reagents, radionuclides, and emergent pollutants, including antibiotic-resistant bacteria, resistance genes, and persistent viruses. The characteristics, volume, and treatment methods for hospital wastewater differ both across and within countries [9].

Despite growing global awareness of the risks associated with hospital wastewater, there remains a significant dearth of empirical data on the microbial and chemical composition of such effluents in North African countries, particularly Algeria. Most existing studies in the region have focused either on physicochemical analyses or limited bacteriological evaluations, with very few addressing both dimensions concurrently. Specifically, the absence of comprehensive assessments integrating microbiological pathogens (e.g., *Escherichia coli*, *Staphylococcus aureus*, *Salmonella* spp.), antibiotic resistance profiling, and toxic trace metal content in Algerian hospital effluents represents a critical gap in environmental and public health research. Moreover, the impacts of such effluents on downstream marine environments and livestock, which are transported through irrigation channels, remain poorly characterized. The lack of published datasets from Algeria contrasts sharply with the situation in other countries, such as Morocco and Egypt, where more structured monitoring frameworks have been employed. Furthermore, no previous studies in Algeria have compared the characteristics of hospital effluent with those of shared urban drainage systems discharging into coastal ecosystems. This lack of monitoring capacity limits the formulation of evidence-based mitigation strategies and hinders the integration of wastewater surveillance into national One Health policies.

This study aims to generate baseline data on the bacteriological and physicochemical characteristics of wastewater discharged from three coastal hospitals in Oran, Algeria, and to evaluate their potential health and ecological risks. Specifically, the investigation seeks to (i) quantify and compare concentrations of key microbial indicators (*E. coli*, *S. aureus*, and *Salmonella* spp.); (ii) assess levels of biochemical oxygen demand (BOD_5_), chemical oxygen demand (COD), dissolved oxygen (DO), and pH as indicators of organic pollution; and (iii) determine the concentrations of hazardous trace metals such as lead (Pb), cadmium (Cd), copper (Cu), and nickel (Ni). In addition, the study compares the effluent profiles of the hospital discharges with those from a shared urban drainage collector that discharges directly into a coastal area. By bridging these data gaps, the study provides the first integrative assessment of hospital effluent pollution in Algeria and offers critical insights into the need for specialized wastewater treatment infrastructure and policy enforcement aligned with the One Health approach.

## MATERIALS AND METHODS

### Ethical approval and informed consent

Hospital administrators (Director of Health and Population of the Wilaya of Oran) approved the sampling process (Approval No. 035-01/23), and this study was conducted in accordance with the current ethical standards, particularly those relating to hospital effluent management and environmental protection.

This study was conducted in accordance with ethical principles throughout the preparation of the results, ensuring a responsible approach to contribute to the understanding and improvement of hospital effluent management practices. The system complies with all applicable laws and regulations governing data collection, analysis, and management. No identifiable or confidential patient or hospital staff information was used in this study. The data used in this research comply with data protection principles. This research is not intended to harm the health, well-being, or safety of individuals or the environment. All protocols and methodologies used were reviewed to minimize the risks associated with handling hospital effluent.

As part of the study, all individuals involved gave their verbal informed consent before any collection or use of their data, in compliance with ethical principles of transparency.

### Study period and location

The study was conducted from January 2023 to February 2024. Oran Province in Algeria has a population of approximately 1.5 million and comprises over 45 healthcare facilities, including 18 hospitals, 27 polyclinics, and more than 25 clinical laboratories. Three coastal hospitals were selected for their extensive healthcare services and broad range of medical specialties. Oran University Hospital (35°41′36″N, 0°38′2″W) is one of the largest hospitals in Algeria, with 43 specialties. The Canastel Pediatric Hospital (35°45’12’’N, 0°48’42’’W) treats all sick children in the province of Oran. Tami Medjebeur Hospital, located in a tourist region (35°44’12’’N, 0°45’19’’W), operates year-round with consistently high patient turnover.

**Figure 1:**
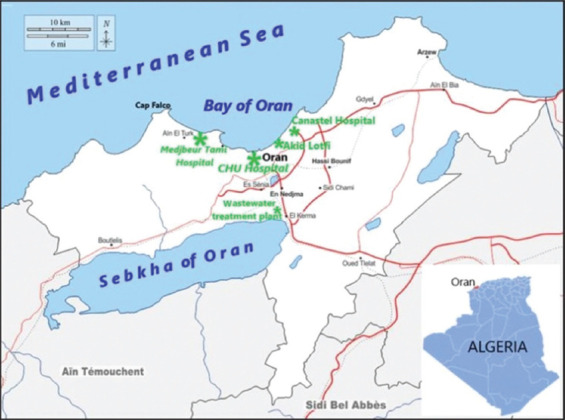
Map of Oran Wilaya showing the locations of three coastal hospital effluents, drain collectors (Akid Lotfi), and wastewater treatment plants (El Kerma) [Source: https://d-maps.com/carte.php?num_car=190688&lang=en].

All three hospitals are in urban areas and discharge their wastewater into a primary urban drainage system that empties untreated wastewater into the sea. A fourth sampling point was established at the drainage outlet immediately before discharge into the sea at 35°43’50 N, 0°35’82’’W. Other healthcare centers in the region are linked to the main urban drain that feeds into the El Karma wastewater treatment plant (Figure 1).

The locations of the three hospitals (Oran University Hospital Center, Canastel Pediatric Hospital, and Tami Medjebeur Hospital), and the corresponding drain collectors (Akid Lotfi) and wastewater treatment plants are shown in Figure 1. The Oran region has a Mediterranean climate characterized by hot, dry summers and mild, wet winters. The average annual temperature was approximately 18°C, with cooler averages in January and February (13°C). Rainfall is less frequent, with an annual average of 350 mm, which explains the tendency toward aridity. These regional climatic conditions are relevant to effluent dynamics.

### Sampling strategy and handling

Direct observation was used to identify peak activity periods on the basis of recurring daily patterns of hospital operations. To capture peak levels of bacterial and chemical contaminants, samples were collected at three specific times at each site; these times are representative of peak contamination during the day. Each partial sample was analyzed in the morning, midday, and afternoon to compare the analyses between the time slots considered representative of peak contamination. The samples were collected monthly from January 2023 to February 2024. Each site was sampled 3 times/day (8:30, 12:00, 16:00) or monthly during the study period. Each site was sampled 3 times/day (8:30, 12:00, 16:00) or monthly during the study period. Each sample consists of three amber glass bottles (Caps & Jars, Netherlands) for bacteriological, chemical, and trace metal analysis. Thus, nine samples and nine replicates were performed per site and per day to correct outliers or missed values. Duplicate samples were collected to ensure analytical reproducibility and reliability. The same number of samples was used for metal traces and chemical analysis.

A small, sterile amber glass bottle (350 mL) was used to measure the bacterial content, following previously described methods by Nuñez and Moretton [10] and Afify *et al*. [11]. To prevent cross-contamination, sterile, powder-free disposable gloves were worn during each sampling event and were replaced between sites. Contamination was minimized by triple-rinsing the bottles and using a uniform sample volume. For quality control, a record of each collected sample was made by carefully labeling each amber glass bottle with a clear, unique sample number. The samples were obtained from beneath the sewage surface through open containers oriented against the current to avoid contamination by surface debris. Bacteria were counted on selective media to determine whether wastewater samples comply with statutory hygiene requirements. For routine quality control, we diluted each bacterial suspension as appropriate and included it within the routine sampling batch.

The samples were transferred into 350 mL sterile containers, transported to the laboratory within 2 h, and refrigerated at 4°C until analysis. To ensure sample traceability, each station was recorded on a record sheet (date, time, and station name).

Sample conditioning during transport is a key step in modifying sample integrity. To preserve sample integrity, biological, chemical, and physical transformations were minimized by transporting samples in coolers with ice packs at 2°C–6°C. All samples were processed and analyzed within 24 h of collection.

### Bacteriological analysis

A sterile inoculation loop was used to transfer microorganisms from one medium to another, such as from an agar plate to a test tube. To prevent contamination, the loop was sterilized before use and was resterilized after each transfer.

To enumerate *S. aureus*, wastewater samples were appropriately diluted, and 0.5 mL aliquots were streaked onto mannitol salt agar plates. The plates were then incubated at 37°C for 24 h. The lower limit of detection observed was 10^3^ *S. aureus* cells within a larger population. Colonies exhibiting yellow fermentation zones were subjected to Gram staining for preliminary identification. Colonies confirmed as Gram-positive cocci were counted using a digital colony counter following established protocols [11–13]. A similar plating method was used to calculate the number of colony-forming units per milliliter (CFU/mL).

Thermotolerant coliforms were analyzed as indicators of *E. coli*, which constitute 80%–90% of this group [14]. The number of *E. coli* bacteria was analyzed using the multiple tube fermentation techniques, in which the bacterial density was estimated using the most likely number method [15]. An alternative method involves membrane filtration through m-ColiBlue24 selective medium (Hach Company, USA), followed by incubation at 44.5°C for 24 h [15]. Sample volumes of 1 mL or 10 mL were used, depending on the expected bacterial loads, in accordance with standard methods.

*Salmonella* spp. was enumerated using bismuth sulfite (CDH, India) as a selective medium, which supports the growth of colonies producing black, diffusible pigments with detection limits ranging from 1 to 10 CFUs/25 g sample [16]. Total coliforms were quantified by preparing serial tenfold dilutions in physiological saline, followed by inoculation into lauryl tryptose broth using Durham tubes (Herbert Durham, India). The tubes were gently shaken and incubated for 48 h at 37°C. Gas production and lactose fermentation in the tubes indicated the presence of positive coliforms.

3M Petrifilm (3M Careers, USA) Select *E. coli* (SEC) Count Plates were also utilized to detect and quantify *E. coli* with a detection limit of 10 CFU/mL, according to the manufacturer’s protocol. A one mL sample was inoculated onto SEC plates and incubated at 44°C for 24 h after being diluted appropriately in 0.1% buffered peptone water (HuanKai Group, China). Regardless of size or color intensity, blue colonies trapped in gas on SEC plates were identified as *E. coli* and enumerated accordingly [14–18].

Pathogenic bacteria in hospital wastewater were identified based on colony morphology, Gram staining, growth on selective media, and confirmatory biochemical tests [15–18]. Culture media specific to the target bacterial strains were obtained from the Pasteur Institute of Oran. The temperatures were carefully controlled during the incubation period. To ensure precision, each sample was analyzed in triplicate by the same operator throughout the study. For quality assurance, known bacterial strains were used as positive controls in the detection assays. Negative controls consisting of sterile solutions were included to confirm the absence of contamination.

For the positive control, we used a duplicate experiment that confirmed the correctness of the tube results of the test. Blank samples were used to identify potential contamination from reagents and equipment. They consist only of water without added analytes. By analyzing the blanks, we can determine whether the method produces any false positive issues from previous samples. Duplicate samples were identical samples of the same material analyzed 3 times within the same run to allow for the assessment of the precision of the analytical method, meaning how close the results were to each other. If results from duplicate samples are significantly different, it indicates that a problem with the analytical procedure is likely to occur. *E. coli* EC958 (Pasteur Insitute, Algiers) was the reference strain used in this study.

### Antibiogram and antibiotic resistance analyses

The most widely used antibiotic susceptibility testing methods are based on the phenotypic detection of antibiotic resistance, which involves measuring bacterial growth in the presence of the antibiotic being tested. The performance of the test was evaluated on strains of *Salmonella* spp. and *S. aureus* in the presence of one of the five most widely used antibiotics in the three hospitals: Methicillin, amoxicillin, amoxicillin + clavulanic acid, ciprofloxacin, and gentamicin, in BD BBL-prepared culture media (BD, USA). Three readings were taken after 24, 48, and 72 h of incubation at 37°C for *Salmonella* spp. and 36°C for *S. aureus*.

### Physicochemical analysis

Wastewater parameters, including temperature, DO, electrical conductivity, and pH, were measured *in situ* using YSI ProQuatro (YSI, USA) multiparameter probes, which were calibrated before each use. The calibration interface of the instrument followed a standardized layout for each parameter, facilitating consistent measurement. According to the YSI protocol, the conductivity was first calibrated because the derived salinity value was used to correct for dissolution.

For chemical assessments, 250 mL wastewater samples were collected monthly from four stations to determine BOD_5_, COD, and DO levels (Figure 1). BOD_5_ was measured through the standard 5-day incubation test at 20°C, following American Public Health Association 5210B guidelines [19]. Samples were incubated at 20°C for 5 days. This temperature is typically maintained within the range of 20°C–25°C to mimic the conditions in which natural microbial populations in freshwater environments thrive, ensuring optimal microbial metabolism and accurate BOD measurements. This test involved diluting each sample with a nutrient buffer, followed by incubation in the dark at 20°C for 5 days. The BOD_5_ was calculated as the difference between the initial and final DO concentrations after incubation. The results are reported in milligrams of oxygen per liter (mg O_2_/L).

The COD represents the oxygen equivalent consumed during the chemical oxidation of organic compounds through the use of a potent oxidizing agent. The digestion method for COD analysis involved oxidizing organic matter in a water sample with an oxidizing agent (potassium dichromate) in acid and measuring the remaining oxidizing agent. Spectrophotometry was used to determine the amount of remaining oxidizing agent. COD was quantified through visible spectrophotometry at 620 nm using a HACH DR/6000 spectrophotometer (Uv Vis Industry Advanced Lab, China). The samples were predigested with 18.1 M sulfuric acid (BASF, Germany) and 0.04 M potassium dichromate (Mubychem group, India) at 150°C for 2 h before analysis. BOD_5_ values were derived by comparing initial and final oxygen concentrations after 5 days of microbial incubation, following standard methods [6, 19, 20]. COD is the amount of oxygen equivalent consumed during the chemical oxidation of organic matter by a strong oxidant. Thus, BOD reflects the biodegradable organic load, whereas COD encompasses both biodegradable and refractory organic substances. The BOD_5_/COD ratio was used to assess the biodegradability index of the wastewater samples; high values indicate high organic matter consumption.

### Trace metal analysis

At each sampling interval (08:30, 12:30, and 16:00), 250 mL of wastewater was collected from the principal drain connected to the three hospitals and from the sea discharge outlet for Cd and Pb analysis; an additional 150 mL was collected for the determination of Cu, zinc, and Ni concentrations. All the sampling bottles used for trace metal analysis were pre-cleaned using the Ultraclean protocol to minimize contamination [19].

To preserve trace metal samples, nitric acid (HNO_3_) was added to the sample container at the time of collection to achieve a pH <2. This acidification helps prevent metal precipitation, adsorption to the container walls, and microbial degradation. The samples used for trace-metal analysis were acidified under a laminar flow hood and stored in coolers to preserve their integrity during transport. Sample preparation for trace-metal analysis involves metal digestion, which aims to destroy the sample matrix by adding an acid (oxidizing agent) and treating it with heat. This process removes unwanted components, leaving only the target analyte and achieving homogenization and pre-concentration. Trace metals were identified and quantified through atomic absorption spectroscopy (SAFAS AA181, Monaco) following sample mineralization and analysis through the dithizone spectrometric method [16, 17, 19, 21]. The SAFAS AA181 spectrophotometer is equipped with high sensitivity, automated gas flow control, and dual background correction capabilities. The instrument determines the concentration of an analyte in a sample and operates within a spectral range of 190 nm–900 nm, offering enhanced sensitivity even at challenging wavelengths (bandwidth settings of 0.2 nm, 0.7 nm, and 2 nm were available for tailored metal detection requirements). The range of the calibration curve was designed to cover the expected concentration range of the analyte in the samples to be analyzed. The detection limits were determined using the calibration curve. For Cd, a calibration curve range might be 0.12 parts per million (ppm)–0.96 ppm if the 1% absorption value is 0.012 ppm. Accurate quantification at low concentrations was achieved by constructing calibration curves using low-concentration standard solutions. All trace metal concentrations are reported as mg/kg fresh weight, equivalent to ppm. Open vessel digestion uses acids to degrade samples in open containers under low pressure and heat on hot plates. Closed vessel digestion involves microwave-assisted acid digestion. This technique involves exposing a sample to a strong acid in a closed vessel and raising the pressure and temperature through microwave irradiation. The reagent mixt used for metal digestion in analytical procedures includes HNO_3_, characterized by strong oxidizing properties and does not produce ionic precipitates. All trace metals and chemical parameter determination were conducted in the chemistry laboratory of Oran University using validated protocols.

### Statistical analysis

Statistical analyses were conducted using Excel Office 365 (Microsoft Office, Washington, USA), with a one-way analysis of variance employed to compare group means. Correlations and p-values are essential statistical tools used to analyze data, draw meaningful conclusions, and determine if there is a statistically significant difference between the means of data. The correlation measures the strength and direction of the relationship between two variables. P-values quantify the probability of observing a result as extreme or more extreme than the one observed, assuming the null hypothesis is true. The mean concentrations and standard deviations for all measured parameters were calculated and evaluated against the WHO guideline values [22]. Each sample was analyzed in triplicate to ensure data reliability and to prevent missing values throughout the study duration. Outliers identified through statistical screening were excluded from the final dataset. Statistical significance was assessed through permutation tests, with 2–3 replicates per sampling station; a p < 0.05 was considered statistically significant. Correlation analyses were performed between the physicochemical and bacteriological parameters, and associations with p < 0.05 were considered significant.

All data from the sample analyses were carefully reported in Excel. Three replicates were used during the study period because three outlier data from the bacteriological analyses were redone to correct these outlier values.

## RESULTS AND DISCUSSION

### Bacterial analysis

**Figure 2:**
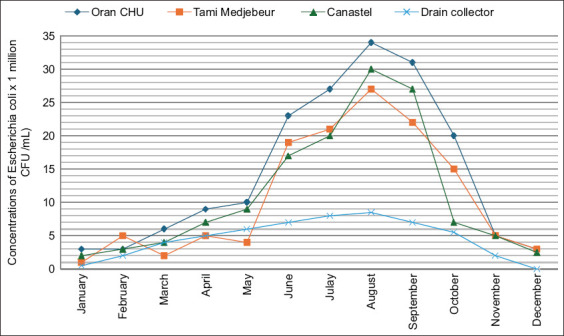
Monthly concentrations of *Escherichia coli* (× 10^6^ CFU/100 mL) from the studied hospital effluents and drain collector. Colony-forming unit, mL=Milliliter.

**Figure 3:**
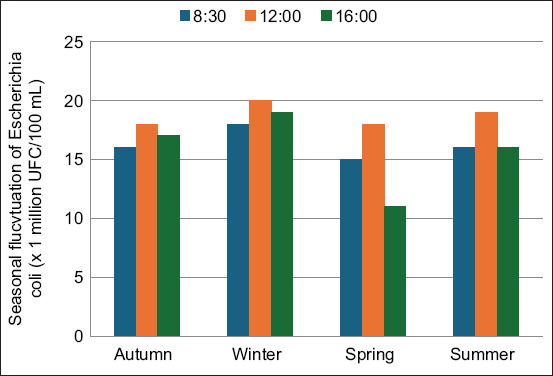
Seasonal fluctuations of *Escherichia coli* (× 10^6^ CFU/100 mL) during high-activity services at Tami Medjebeur Hospital effluent. Colony-forming unit, mL=Milliliter.

**Figure 4:**
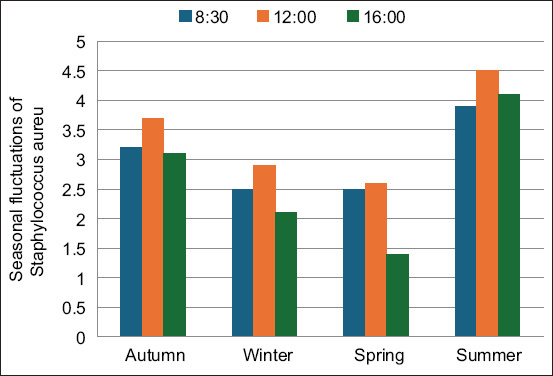
Seasonal fluctuations in *Staphylococcus aureus* concentrations (×10^6^ CFU/100 mL) during high activity of hospital services at Oran UHC. CFU=Colony-forming unit, mL=Milliliter.

The microbiological characterization of hospital wastewater is crucial for evaluating the environmental and public health implications of effluent discharge from healthcare facilities. The microbiological findings are presented in Figures 2-4.

The average *E. coli* concentration in hospital effluents ranged from 0.5 × 10^6^ to 34.5 × 10^6^ CFU/100 mL. These values exceed those reported in hospital effluents [23–25]. Fekadu *et al*. [23] reported concentrations of 2.10^3^–2.10^6^ fecal coliforms/100 mL in the effluent of a hospital in Lyon City (France).

Coliform levels serve as key indicators of fecal contamination and may indirectly reflect the presence of residual antibiotics and disinfectants. These coliform concentrations are often used as indicators of water pollution levels by fecal bacteria and are considered indirect indicators of the presence of antibiotics and disinfectants. Medicines reach hospital effluents through patients’ urine, stools, and various biological fluids (such as sweat, saliva, and vomit) or through the disposal of unused or expired medicines through sewers and sinks. The use of disinfectant and antiseptic products may be insufficient for not only healthcare activities but also cleaning products, particularly those containing chlorinated compounds, which are preferred [23].

Despite high water consumption, dilution along internal drainage pathways does not sufficiently reduce bacterial concentrations in the main hospital effluent. The reduction in the concentration of various bacterial species is insufficient to dilute the wastewater.

The monthly fecal bacteria load values recorded in the three effluents were greater than those recorded in the drain collector (Figure 4). These findings align with those of previous studies by Majumder *et al*. [2] and Emmanuel *et al*. [5], which have shown that hospital effluents generally have higher bacterial loads than urban wastewater. These results are consistent with those of Ameziane and Benaabidate [24], which indicate that the effluents of health establishments are generally less loaded with microbiological contaminants than urban wastewater.

The bacterial concentrations peaked between June and September, likely due to the promotion of microbial proliferation by elevated temperatures and organic loads. The increase in pollutant concentration is related to temperature and the relatively high organic load in summer, which favors bacterial growth in the wastewater environment. On the other hand, there is a lack of precipitation which can also be a concentrating factor in the winter and spring periods and consequently reduce the concentration of coliform bacteria.

*S. aureus* concentrations ranged from 1.5 × 10^6^ to 4.5 × 10^6^ CFU/100 mL across the sampled effluents (Figure 4). These values are higher than those reported previously by Majumder *et al*. [2], Ameziane and Benaabidate [24], and Chitnis *et al*. [25] (1.2 × 10^5^ CFU/100 mL, 2.4–6.08 × 10^2^ CFU/100 mL, and 2.5 × 10^5^ CFU/100 mL, respectively). This may reflect inadequate water use or insufficient application of cleaning agents and disinfectants in hospital departments.

*S. aureus* strains were resistant to the five antibiotics tested, exhibiting significant resistance. In addition, the presence of resistant bacterial strains suggests possible adaptations to antibiotics and disinfectants commonly used in clinical settings [10, 24]. *S. aureus* has a strong adaptive capacity and thus acquires various types of resistance to antistaphylococcal agents [13, 14]. *S. aureus* is a pathogen whose strong adaptive ability enables survival through the successive acquisition of antibiotic-resistance genes, mechanisms for regulating growth in the presence of antibiotics, and specific virulence factors [14].

*Salmonella* spp. were detected in all three hospital effluents, primarily during the spring and summer months, with a mean *Salmonella* spp. count of 3 ± 1.46 in Oran University Hospital Center, a mean count of 2.3 ± 1.56 in Tami Hospital, and a mean count of 1.5 ± 1.32 in Canastel Hospital effluent. The number of samples analyzed was n = 9.

*Salmonella* spp. are highly resistant to the antibiotics commonly used in hospitals. The occurrence of these pathogenic bacteria may be attributed to their resistance to antibiotics or disinfectants, as noted in earlier reports by Nuñez and Moretton [10] and Awodele *et al*. [26]. *Salmonella* spp. are resistant to antibiotics such as ampicillin, ciprofloxacin, gentamicin, tetracycline, and third-generation cephalosporins, including ceftriaxone and cefotaxime. One of the key mechanisms through which *Salmonella* biofilms develop AR is efflux [13, 26].

Hospital wastewater acts as a reservoir for emer-ging pollutants, including antibiotic residues and multidrug-resistant bacteria [27–29]. For this reason, antibiotic usage can pose potential environmental hazards and promote antibiotic resistance, and therefore, it should be closely monitored. On-site chlorination contributes to effective microbial disinfection of hospital effluents [30].

Furthermore, Azuma and Hayashi [31] noted the potential effects on public health and the environment that may result from the co-treatment of hospital wastewater with domestic wastewater in treatment plants, as well as the use of the resulting sludge in agriculture. This cotreatment and subsequent agricultural reuse of sludge, recently observed in Algeria, raise environmental and public health concerns [31].

High temperatures can directly affect the distribution, transmission, and persistence of pathogens in the environment, thereby influencing the incidence and spread of climate-sensitive infectious diseases. People and livestock can be infected by ingestion of contaminated water or food, skin contact, or inhalation of water droplets. Infection risks are associated with bacteria such as toxin-producing *E. coli*, *Salmonella* spp., and *Campylobacter* spp. [30, 31].

### Physicochemical characterization of effluents

**Figure 5:**
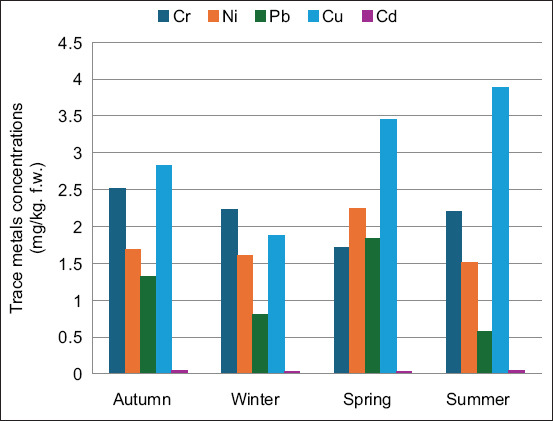
Seasonal concentrations of the trace metals (Cr, Ni, Pb, Cu, and Cd) recorded at the Oran CHU. Cr=Chromium, Ni=Nickel, Pb=Lead, Cu=Copper, Cd=Cadmium.

**Figure 6:**
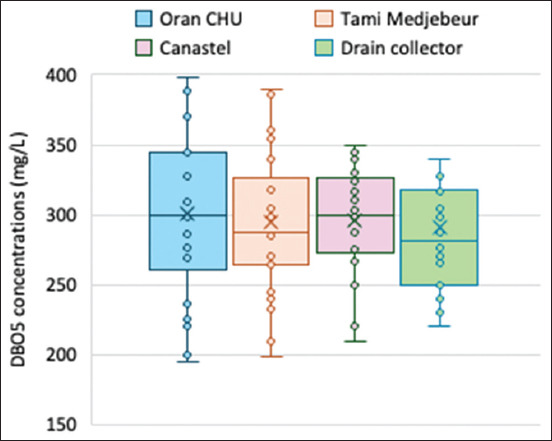
Biochemical oxygen demand concentrations recorded in three hospital effluents and drain collectors in 2023.

**Figure 7:**
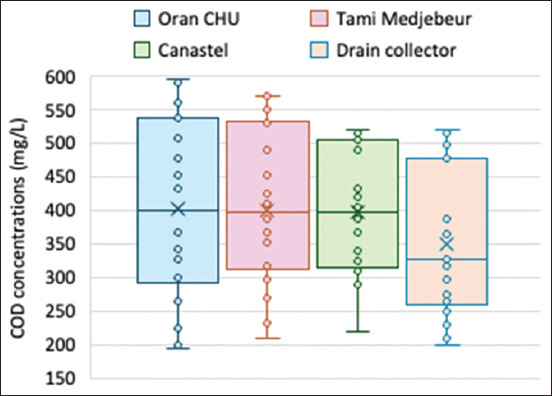
Chemical oxygen demand concentrations recorded in hospital effluents and drain collectors during 2023.

The physicochemical parameters, including temperature, conductivity, pH, DO, BOD_5_, and COD, are presented in Figures 5-7. The effluent temperatures from the three hospitals and the collector were 20.6°C and 29.6°C, respectively. The mean temperatures were 24.68°C at OUHC, 24.51°C at Canastel Pediatric Hospital, 24.85°C at Tami Medjebeur Hospital, and 25.50°C in the collector. Water temperature directly influences the physicochemical properties and modulates the rate of chemical reactions within the effluent. The wastewater temperature in the drain collector effluent was usually slightly higher at the time of sampling. This could also be due to various hospital activities of different services, such as sterilization of instruments, cooking, and showering, which all use hot water.

The electrical conductivity is an indicator for estimating the overall mineralization, evaporation, and total amount of water-soluble salts in hospital wastewater. The electrical conductivity of hospital wastewater ranged from 250 μS/cm to 1,353 μS/cm, with an average of 755 μS/cm ± 490 μS/cm recorded in the effluent from the Oran University Hospital Center, followed by the drain collector with 721 μS/cm ± 338 μS/cm. These elevated conductivity values suggest a high concentration of dissolved salts and ions, which may impair water quality. The values obtained for most hospital effluent samples were below the WHO recommendations (2000 μS/cm) and were exposed to evaporation in hot weather, indicating highly mineralized water [32]. These values indicate that wastewater is rich in mineral salts and ions, which can interfere with water quality.

The pH values across all hospital sites and the collector were nearly neutral, ranging from 6.5 to 7.8 (7.66 ± 0.46) and the pH levels remained relatively stable throughout the study duration at all sampling sites. The pH remained neutral to slightly basic throughout the study period, with similar variations observed at all four stations. The values fell within the WHO-recommended pH range of 6–8.5 [3].

The mean DO concentrations in both the hospital and collector effluents were 2.5 mg/L–4.5 mg/L. The mean DO concentrations recorded at the four stations during the monthly sampling were as follows:

3.04 ± 1.42 mg/L Oran CHU effluent;
2.56 ± 1.70 mg/L Tami Medjebeur Hospital effluent;
2.1 ± 1.85 mg/L Canastel Hospital effluent;
2.09 ± 1.68 mg/L in the drain collector.

The maximum value was recorded in Oran CHU at < 4.44 mg/L and remained below the WHO standard (>5); this indicates that the effluents are loaded with organic matter and bacteria, which consume a significant quantity of DO, facilitated by relatively warm temperatures, especially in the summer.

### Quantification of trace metals in wastewater

The trace metal concentrations varied depending on the sampling location, time of day, and type of metal. The analysis revealed contamination of all hospital effluents and the main collector with at least four toxic metals. Pb, Ni, Cu, and Cd were the most frequently detected trace metals in the effluents (Figure 5). The average concentrations were 0.064, 0.057, 0.17, and 0.30 mg/kg for Pb, Cd, Ni, and Cu, respectively.

Higher concentrations of heavy metals were recorded during the summer months than during the other seasons. This increase may be attributed to intensified hospital activity and increased use of chemical reagents during the summer, such as radiographic agents, disinfectants, and acids. The effluent discharge of heavy metals poses ecological risks to aquatic organisms and potential public health hazards through bioaccumulation in food webs [33]. Lead concentrations varied across sampling sites but consistently exceeded the permissible discharge limits. Cd levels were particularly elevated in samples from Canastel Pediatric Hospital. Ni and Cu were more concentrated in the effluents from OUHC compared with those from the other two hospitals.

Except for Cd, all measured concentrations exceeded the WHO guideline limits for effluents discharged into aquatic environments (Table 1 and Figure 5) [22, 33, 34]. Compared to the results of Bennama *et al*. [33] from the Oran urban wastewater treatment plant of El Karma, the concentrations of trace metals in Table 1 are generally higher in most cases.

**Table 1:** Average trace metal concentrations (mg/kg f.w.) obtained from monthly sampling at the four studied stations.

Parameters	Oran CHU	Tami Medjeb. Hospital	Canastel Hospital	Drain collector	WHO/FAO standards [22]
Cr	2.17 ± 0.87	1.32 ± 1.08	1.54 ± 0.82	1.34 ± 0.91	≤1.0
Cd	0.04 ± 0.02	0.05 ± 0.05	0.03 ± 0.02	0.04 ± 0.03	≤1.0
Pb	1.64 ± 0.05	0.84 ± 0.31	1.04 ± 0.31	1.03 ± 0.61	≤0.5
Ni	1.77 ± 0.16	1.35 ± 0.25	1.38 ± 0.15	0.86 ± 0.46	≤2.5
Cu	13.84 ± 5.26	9.84 ± 6.54	11.12 ± 6.34	8.95 ± 4.54	-
Zn	14.04 ± 7.58	10.03 ± 5.68	8.73 ± 3.36	12.18 ± 6.37	≤2

The number of analyzed samples=12/station; f.w=Fresh weight. Cr=Chromium, Cd=Cadmium, Pb=Lead, Ni=Nickel, Cu=Copper, Zn=Zinc, WHO=World Health Organization, FAO=Food and Agriculture Organization

These concentrations are likely attributable to the common use of materials for activities in various units, including pipework and internal drainage structures. The presence of trace metals in hospitals may also be associated with various procedures, including radiology, the use of broken equipment (such as thermometers), blood pressure cuffs, fluorescent lights, batteries, and analytical chemicals, as well as medications passed through patients.

Heavy metals present in hospital effluent can pose a threat to human health and the environment due to their excessive concentrations compared to the WHO limits for crop irrigation and marine resources. An adequate purification system is therefore needed to reduce these high levels [22]. The impact of trace metals on human health and the environment has been widely investigated [34], and researchers have proposed new therapeutic approaches to counter their toxicity.

Toxicity may be amplified by synergistic effects when multiple heavy metals co-occur in effluents. These metals are non-biodegradable and tend to persist in aquatic systems, accumulating in sediments and biota. Chronic exposure to these contaminants may lead to toxic effects in marine organisms and endanger livestock consuming vegetation irrigated with contaminated water. The observed seasonal trends underscore the importance of continuous monitoring and stricter enforcement of regulations. Elevated temperatures can intensify the release of trace metals into the environment and accelerate the breakdown of soil organic matter, thereby releasing heavy metals that were previously bound to it. Additionally, increased temperatures can lead to changes in precipitation patterns, which in turn can affect the leaching and transport of heavy metals into water bodies [34].

The values of BOD_5_ concentrations recorded in hospital effluents and drain collectors (Figures 6 and 7) varied annually from 190 mg/L to 400 mg/L, whereas the COD values ranged from 195 mg/L to 600 mg/L. The BOD_5_ values of the collectors ranged from 220.53 mg/L to 370.35 mg/L, which were slightly lower than those of the hospital effluents. The elevated BOD_5_ values during summer were likely due to increased microbial activity facilitated by increased ambient temperatures. BOD_5_ levels were higher in hospital effluents than in the collector, with values ranging from 190.33 mg/L to 398.25 mg/L. The average COD/BOD_5_ ratio across all stations was approximately 2.3. The increase in BOD_5_ and COD concentrations reflects high organic pollution in all these effluents.

COD levels ranged from 200 mg/L to 590 mg/L in hospital effluents and from 220 mg/L to 550 mg/L in collectors. The COD/BOD_5_ ratio and values <2.5 suggest a considerable organic load in the effluents, indicating the partial biodegradability of the wastewater. However, studies by Todedji *et al*. [19] and Bennama *et al*. [33] have yielded higher concentrations than those reported in this study.

In this study, the values of conductivity, BOD_5,_ and COD corresponded with low DO levels and oxygen saturation, indicating high loading of dissolved organics and particulates. The low oxygenation of effluents is likely caused by the high consumption of DO by aerobic bacteria for the degradation of organic matter. This can lead to anoxia and the growth of anoxic bacteria, resulting in reductive and asphyxiating conditions.

Hospital wastewater contains a significant amount of organic matter, specifically COD and BOD_5_, which deplete DO in water bodies. The biological treatment of wastewater is a reliable, affordable, and effective method that produces safe results [35].

Average concentrations of pathogenic bacteria, trace metals, and physicochemical parameters indicate a concerning situation for both human health and the environment. Evaluation of the quality and effective-ness of wastewater treatment at these hospitals is essential [36, 37] because the presence of high bacterial loads, toxic chemicals, and microbiological compounds must be reduced.

Consequently, further fieldwork, supported by hospital managers, is needed to help optimize treatment plant efficiency and facilitate the implementation of circular sludge management plans, which remain unexplored [38–41]. Green management plans for hospital institutions should be considered for the three hospitals reported here, not only to increase efficiencies in materials usage and climate change adaptation, but also to reduce biological hazards and threats, including the emerging problem of antibiotic resistance. Such plans are being rapidly undertaken in many countries, and many examples exist (e.g., https;//royalmarsden.nhs.uk/about-royal-marsden/quality-and-safety/regulatory-information/our-green-plan#). Policies and planning are essential to help the treatment of people (staff and patients) and improve resources, and enabling proper treatment of hospital waste is crucial to mitigate the long-term effects on human health and the surrounding ecosystems.

Compared with those reported from North African countries, Egypt [11], Africa (Ethiopia [7, 23] and Benin [19]) and Morocco [24, 27], our findings on pathogen bacteria and trace metal toxicity also threaten public health and the environment, with an urgent recommendation for adequate and effective treatment of hospital liquid discharge.

The discharge of untreated hospital wastewater raises major concerns in the context of “One Health” due to the risks of contamination and the spread of zoonotic diseases, including livestock. This water can contain human and animal pathogens, as well as drug residues, which can contaminate the environment and cause diseases in humans and animals.

## CONCLUSION

This study assessed the chemical and bacteriological characteristics of effluents discharged from three coastal hospitals in Oran, Algeria, as well as their associated drainage collectors that discharge into the Akid Lotfi marine ecosystem. The findings revealed elevated concentrations of fecal coliforms, *E. coli*, *S. aureus*, and *Salmonella* spp. in all samples, with peak microbial loads observed during the summer months. The effluents consistently exhibited high levels of toxic trace elements and pathogenic or potentially pathogenic bacteria, particularly between 8:30 a.m. and 12:30 p.m., coinciding with peak hospital activity. Trace metal analysis confirmed that concentrations of Pb, Cd, Ni, and Cu exceeded the WHO effluent guidelines, raising significant concerns regarding environmental persistence, bioaccumulation, and associated toxicity. Seasonal elevations in heavy metal concentrations may be attributed to intensified hospital operations, climatic variability, and the increased use of chemical reagents.

These findings underscore the critical need for dedicated treatment systems for hospital liquid waste to mitigate the transmission of infectious diseases. Microbial contamination of this magnitude presents serious public and veterinary health risks, particularly to livestock that graze on vegetation irrigated with untreated wastewater. The analysis of BOD_5_, COD, and DO concentrations indicated substantial organic loading in hospital effluents, suggesting the presence of both biodegradable and recalcitrant organic pollutants. Trace metal concentrations were notably higher in the spring and summer months, likely due to elevated water temperatures and intensified clinical activities. To address these challenges, the implementation of electrocoagulation reactors and advanced oxidation processes is recommended to effectively remove organic pollutants, microbial contaminants, and viral particles from hospital effluents.

In aquatic systems where no prior treatment is applied – a common occurrence in Algeria – these pollutants and pathogens compromise environmental integrity far beyond the immediate hospital vicinity, facilitating the spread of waterborne diseases. Given the considerable toxicity and pollutant load, wastewater management strategies must be tailored to the specific discharge characteristics of each healthcare facility. The findings highlight the significant role of medical centers in polluting coastal soils and contributing to the transmission of infectious diseases in marine ecosystems. This study further advocates for regular monitoring of the biological and ecological conditions of marine environments receiving hospital discharges to determine the required purification strategies for each institution.

In view of the critical environmental threat posed by untreated hospital wastewater, the urgent development and implementation of specific treatment systems are paramount to elevate environmental protection standards. Continued research and systematic monitoring are crucial for controlling and mitigating the risks associated with microbial contamination in healthcare effluents. The deployment of appropriate treatment technologies and adherence to stringent regulatory frameworks are vital to safeguarding public health and ecosystem sustainability [40, 41]. The current state of hospital effluent quality necessitates immediate action to establish effective pre-discharge treatment infrastructure. In particular, measures must be taken to restrict livestock access to vegetation irrigated by wastewater from treatment plants such as El Karma, given the potential for microbial and heavy metal contamination.

Overall, the findings of this study emphasize the urgent need for healthcare facilities to adopt dedicated wastewater treatment protocols. Routine environmental surveillance and the enforcement of legally binding discharge standards for hospital effluents are critical. The continued discharge of untreated hospital wastewater significantly contributes to aquatic pollution and poses a severe risk for the propagation of antimicrobial resistance and ecological degradation. Future research should investigate the long-term impacts of hospital wastewater disposal on coastal ecosystems and human health.

## AUTHORS’ CONTRIBUTIONS

SH: Conceptualization, methodology, sample collection, and manuscript drafting. DB: Validation and formal analysis, data curation, and investigation. RF: Physicochemical and trace metals data interpretation and reviewed and edited the manuscript. All authors have read and agreed to the publication of the final version of the manuscript.
